# Massive malignant phyllodes tumor accompanied by anemia and ulceration in the breast: A case report

**DOI:** 10.1002/ccr3.9096

**Published:** 2024-06-16

**Authors:** Xiaoxiao Dong, Dong Song, Jinxiu Ma, Jian Sun, Xiaozhen Wang

**Affiliations:** ^1^ Department of Breast Surgery, General Surgery Center The First Hospital of Jilin University Changchun Jilin China

**Keywords:** breast cancer, complications, diagnosis, malignant phyllodes tumor, pathology

## Abstract

Large malignant breast phyllodes tumors are uncommon in clinical settings. Here, we report such a case to provide a reference for clinical work. A 48‐year‐old woman identified a lump in her right breast, which eventually grew up to 25 cm × 10 cm and began to rapidly bleed and ulcerate within 3 months. The patient had visible signs of anemia and significant emaciation as a result of the tumor's wasting effect and the protracted course of the disease. The patient underwent a modified radical mastectomy on the right breast. The pathology results obtained after surgery revealed a malignant phyllodes tumor. No adjuvant therapy, such as chemotherapy or radiation, was administered. The patient had no symptoms of tumor recurrence and complications from the surgery after a follow‐up of 9 months.

## 1 INTRODUCTION

Phyllodes tumors of the breast (PTB), formerly known as “cystosarcoma phyllodes,” are relatively rare in clinical practice, and they account for 0.3%–1% of breast tumors.^1^ According to the histological features, the World Health Organization currently divides PTB into three categories, namely benign, borderline (also known as low‐grade malignant PTB), and malignant (also known as high‐grade malignant PTB).^2^ PTB rarely affects men, and typically affects women between 40 and 50 years of age.^3^ In most cases, the clinical signs are unilateral, typically exhibited as a painless mass, and there may be a history of quick growth.

The high‐risk factors and pathogenesis of PTB are currently unclear. There are hypotheses suggesting that PTB originates from fibroadenomas, but there is still a significant debate.^4^ High estrogen status may also be an independent pathogenic factor for PTB.^5^ This tumor is a fibroepithelial tumor that contains both stromal and epithelial components. Reduction in the epithelial component is associated with greater malignancy. The characteristics of phyllodes tumors are composed of proliferative stroma accompanied by elongated fissure‐like gaps, with the surface of the gaps covered by epithelium. Chromosomal changes are associated with the malignant phenotype of PTB. In borderline and malignant PTB, chromosome 1q amplification is common, and as the degree of amplification increases, malignant behavior increases.^6^, ^7^

Phyllodial breast tumors are generally rare; however, their incidence rates have increased in recent years. The clinical manifestations of phyllodial tumors lack specific characteristics but include insidious onset, slow progression, a long medical history, and the main manifestation being rapid growth of painless masses in the affected breast. These characteristics make it difficult to determine the nature of phyllodial tumors without surgery. In the present study, we report a case of a giant malignant phyllodial tumor and emphasize the importance of “detect, diagnose, and treat early” to avoid serious complications.

## 2 CASE PRESENTATION

### 2.1 Background of the case

A 48‐year‐old female was admitted to our hospital with a right breast tumor. Six years ago, the patient identified the tumor in her right breast by chance. The tumor was left untreated and showed no signs of redness, swelling, pain, or ulceration. Three months ago, following a COVID‐19 infection, the right breast's lump rapidly grew, accompanied by bleeding and ulceration but without purulent secretion.

### 2.2 Physical examination and laboratory tests

**FIGURE 1:**
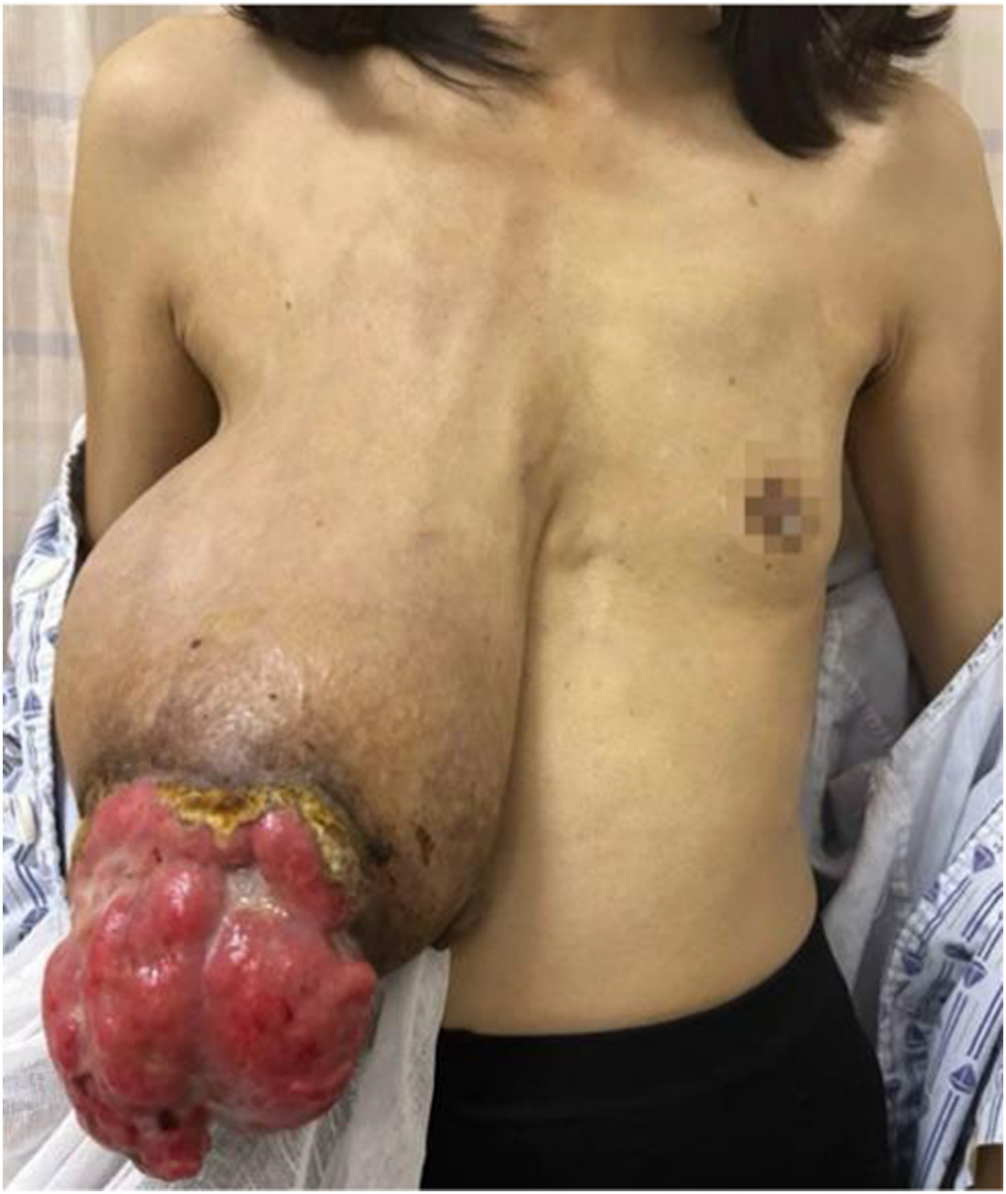
Preoperative anterior view of the patient with phyllodes tumors of the breast.

**FIGURE 2:**
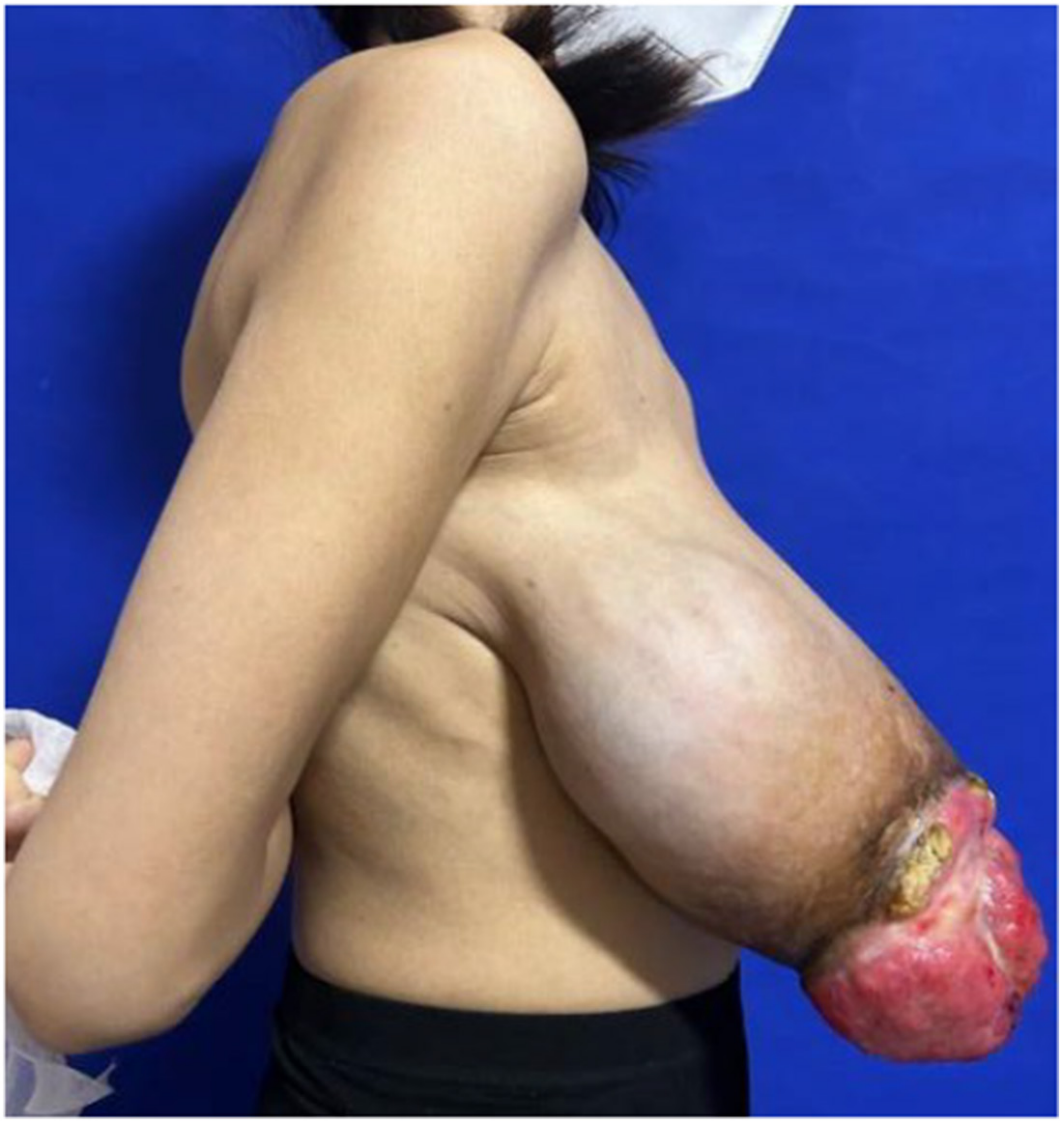
Preoperative lateral view of the patient with phyllodes tumors of the breast.

**FIGURES 3:**
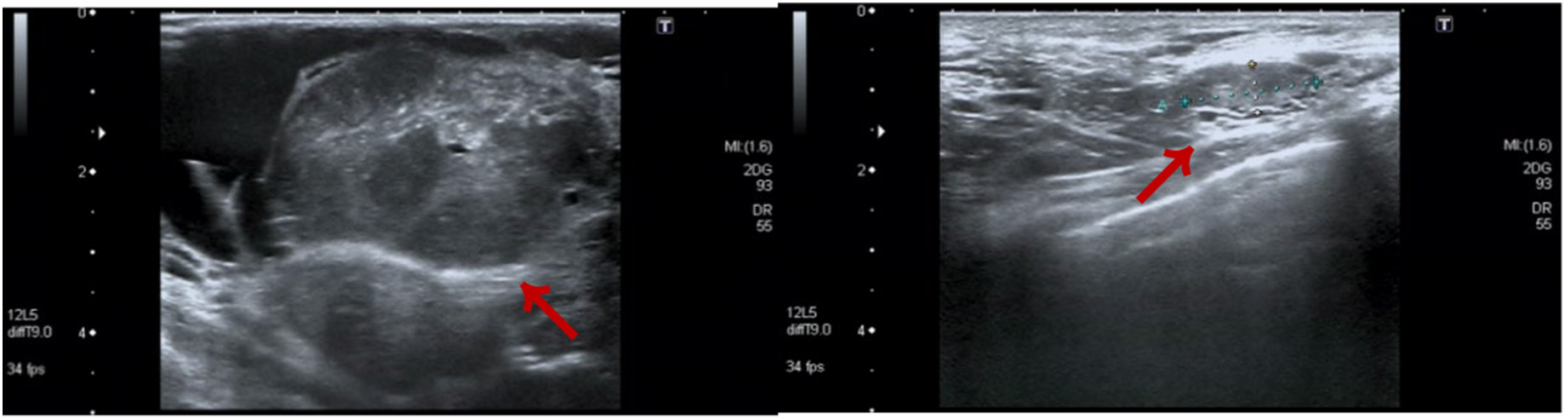
Color ultrasonography evaluation of the patient before surgery.

Physical examination of the patient revealed pale nail beds on both hands, pale eyes, and an anemic face. The skin on the right breast's lateral quadrant was pigmented, with surface ulceration and bleeding, and the right breast was noticeably bigger than the left one. The left breast showed no signs of skin redness, swelling, nipple depression, dimples, or orange peel sign. The right breast was noticeably enlarged, and the tumor measuring roughly 25 cm × 10 cm protruded from the skin (Figures 1 and 2). The left breast did not have any discernible bulk. There were no swollen lymph nodes palpable in the bilateral supraclavicular area or left armpit, but there was a lymph node measuring roughly 2 cm × 1 cm in the right armpit. Ultrasound examination revealed a mixed echogenic mass of approximately 20 cm × 5.2 cm on the right breast. Blood flow signals were present within the bulk, and the boundary was clearly defined despite the uneven shape. In addition, ultrasound indicated hyperplastic alterations in the left breast. The left axillary region did not appear to have any noticeable anomalous lymph nodes. The right armpit revealed many lymph nodes, and the largest one was 1.6 cm × 0.6 cm in size and had a thicker cortex compared with the normal cortex. Ultrasonography of the right breast tumor indicated that the lesion was BI‐RADS4 class 4b (Figure 3). Positron emission tomography–computed tomography was also performed, and the right breast mass could not be ruled out as a phyllodes tumor with malignant transformation due to (1) its heterogeneous metabolism and mixed density; (2) elevated metabolism of lymph nodes situated in the right axilla and behind the pectoralis major and minor muscles, possibly suggesting the presence of metastasis; (3) slight inflammation in the upper and lower lobes of both lungs; an inflammatory small nodule in the upper lobe of the right lung (we consider this is related to COVID‐19). Puncture pathology of the right breast fibroepithelial tumor did not show any conclusive indications of malignancy. Because fibroepithelial tumors are heterogeneous and biopsy tissue is limited, it is important to integrate clinical information and, if necessary, perform full lesion resection. The blood routine results indicate that hemoglobin is 75 g/L and white blood cell count is 13.68 × 109/L.

### 2.3 Treatment

**FIGURE 4:**
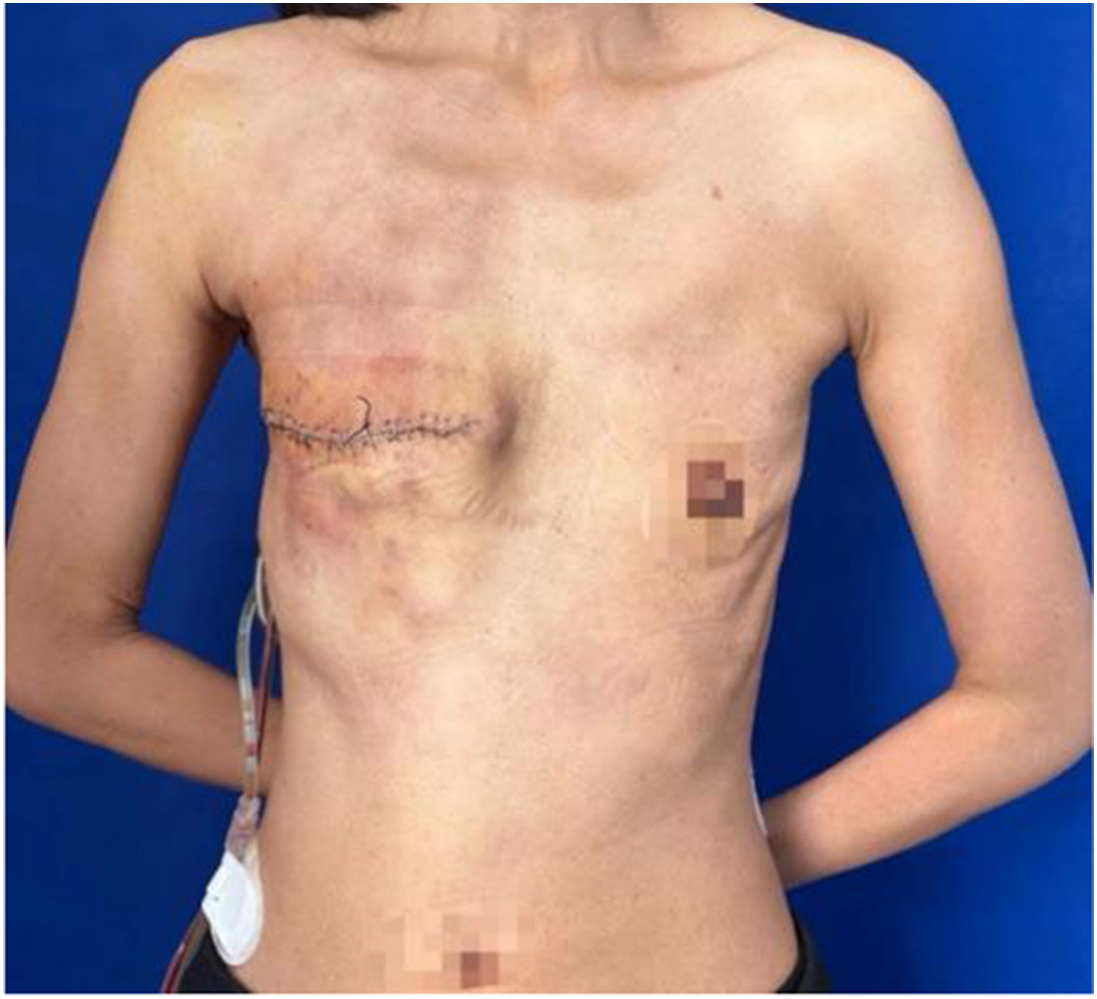
Postoperative anterior view of the patient with phyllodes tumors of the breast.

Preoperative diagnosis was a right breast phyllodes tumor (high likelihood of malignancy), and right axillary lymph node metastasis was highly probable. Owing to the tumor's massive size and ulceration, the patient's blood routine test revealed mild anemia and infection. Hemoglobin level reached 99 g/L following a 400‐mL leukocyte‐free suspended red blood cell transfusion and using antiphlogistic drugs to treat inflammation. A modified right breast radical mastectomy was performed after the patient's status was found to be stable (Figure 4). About 40 mL of blood was lost during the successful procedure.

**FIGURE 5:**
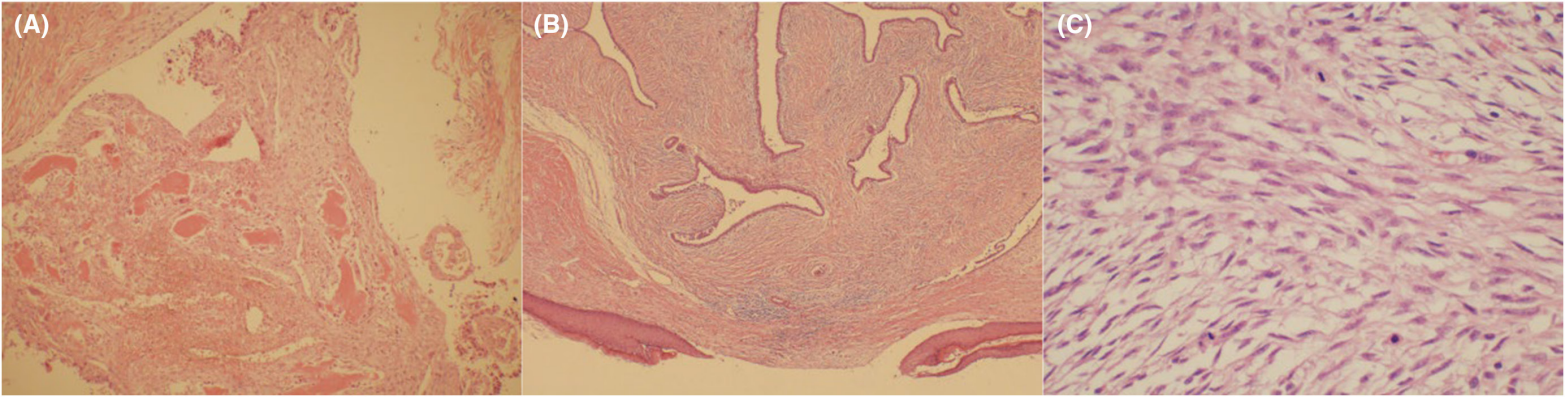
(A) Tumor cell–rich area, with mat striate structure (HE × 100); (B) lobular tumor invading skin and subcutaneous tissue, leading to the formation of skin ulcers (HE × 40); (C) tumor cell nuclei are slightly isoplastic, nuclei are spindle or spindle shaped, and nuclear division images are seen (HE × 400).

General morphology of surgical specimens: There was a large lobulated mass in the breast tissue (22 cm × 21 cm × 7.5 cm), with a solid‐and‐tough cut surface. The total area of the breast and axilla measured 24 cm × 23 cm × 8 cm; the area of the fusiform skin measured 24 cm × 17 cm; and the diameter of the nipple was 1.8 cm. The breast did not contain any normal glands. The tumor was located 0.1 cm from the superficial fascia. Locally, the tumor affected the skin. A single adipose tissue mass of 8 cm × 7 cm × 2 cm was discovered, and 25 lymph nodes with a diameter of 0.3–2 cm were palpable inside of the mass. Microscopic examination (Figure 5) showed a 22 cm × 21 cm × 7.5 cm malignant phyllodes tumor on the right breast. The interstitial sarcoma component is low‐grade fibrosarcoma, and the tumor part shows myofibroblastic differentiation, focal ossification, and penetration into the duct to form a papilloma‐like morphology. The tumor lacked a distinct capsule, most of which exhibit expansion and progressive growth, local skin invasion, and formation of skin ulcers. The tumor cells showed a wide range of morphologies, including myxoid, sparse, dense, and collagenized cells and presented mild‐to‐severe atypia. No tumor metastases were found in the axillary lymph nodes, and there was no tumor infiltration of the papilla or superficial fascia (0/25). The postoperative pathological diagnosis was a malignant breast phyllodes tumor.

### 2.4 Outcome and follow‐up

After surgery, the patient recovered fully. Half a month after the operation, hemoglobin level improved (92 g/L), and no adjuvant therapy, such as chemotherapy or radiation, was administered. At a 9‐month follow‐up after surgery, the patient has not experienced any associated problems or indications of tumor recurrence thus far. In addition, the patient's hemoglobin remained stable at 100–110 g/L, and her physical condition has significantly improved.

## 3 DISCUSSION

Cases of massive malignant PTB with rupture and anemia are extremely rare. At present, there is no clear definition of giant PTB, but it is generally considered that tumors larger than 10 cm are giant PTB. Patients with giant PTB, especially those with malignant giant PTB, have many complications and significant adverse reactions. Due to the rapid tumor growth in patients with massive malignant PTB, varicose veins can form on the skin surface, which can lead to necrosis and infection due to insufficient blood supply, resulting in ulceration. Tumor rupture and vascular rupture can lead to bleeding. Patients with malignant PTB may experience significant systemic signs such as anemia and significant emaciation due to the consumption effect of tumors or concurrent infection and bleeding.^8^ In severe cases, cachexia may occur, leading to systemic failure. In this case, the patient's mass significantly and rapidly increased, with visible varicose veins, bleeding, and infection on the surface of the skin. The patient also exhibited symptoms such as decreased appetite, significant emaciation, and anemia, all of which are consistent with the characteristics of malignant PTB.

Mammography and color Doppler ultrasound are the two primary imaging tests for diagnosing PTB. However, in the diagnosis of early PTB, breast ultrasonography and mammography frequently do not show particular signs.^9^ For most cases of PTB, excision biopsy is needed for a definitive diagnosis because the condition's early imaging and histological signs are similar to those of breast fibroadenoma, which limits the diagnostic significance for clinicians and makes it difficult to make a decision before surgery. Divergent opinions exist on the ability of MRI to diagnose this illness. Certain aspects of MRI may aid in differentiating PTB from fibroadenoma, according to the Chinese expert consensus on the diagnosis and treatment of breast lobulated tumors in women. While PTB is more likely than fibroadenoma to have internal cystic alterations and increased signal intensity of breast tissue surrounding the lesion on T2WI, there are no discernible differences among various types of PTB.^10^ It is challenging to diagnose PTB with imaging tests prior to surgery. PTB must be distinguished from breast sarcoma and breast fibroadenoma. 1. Breast fibroadenoma: Dot calcification is observed in mammography, and the mass is primarily shaped like an ovoid or sphere with a smooth surface and leathery texture.^11^ Histologically, it is primarily made up of proliferating fibrous stroma and glands. The surrounding fibrous connective tissue may compress the round or ovoid glands into slit‐like shapes, but they lack PTB's phylloidal growth pattern and low mitotic rate. 2. Breast sarcoma: This is an uncommon kind of malignant tumor that arises from the stroma and is related to angiosarcoma^12^ and fibrosarcoma. Typically, it takes the form of an oval, hypoechoic mass with an unclear border.

Surgery is the first line of treatment for PTB; however, the specific surgical technique is still highly debatable. With her full consent, the patient underwent a complete mastectomy due to the large PTB. Nevertheless, nearly every study conducted to date has demonstrated that patients who have had a complete mastectomy and those who have had an extended local mastectomy do not significantly differ in terms of overall survival or cancer‐specific survival. The most recent consensus among Chinese experts regarding the diagnosis and management of women's breast lobulated tumors states that patients with borderline or malignant PTB should first undergo extended local excision. According to National Comprehensive Cancer Network (NCCN) guidelines, patients with borderline and malignant PTB should have a margin of at least 1 cm for local extended resection.^13^ The resection margin was found to be associated with the recurrence of phyllodes tumors in a study by Mangi et al.;^14^ pertinent reports have also shown that there is no significant difference in the prognosis between total mastectomy and breast‐conserving surgery when there is no distant metastasis and the resection margin is less than 1 cm.^15^ An independent predictor of better disease‐free survival and a lower rate of local recurrence are a negative resection margin. For individuals with PTB, ensuring a negative resection margin is advantageous. It is unclear how patient age, tumor size, and histological grade relate to a negative resection margin.^16^, ^17^, ^18^, ^19^ Although axillary lymph nodes are frequently enlarged in patients with PTB, axillary lymph node dissection is rarely advised because a small percentage of patients (<5%) have axillary lymph node metastases.^20^ Due to the preoperative suspicion of axillary lymph node metastases, our patient underwent axillary lymph node dissection. However, none of 25 lymph nodes had tumor metastases, according to the postoperative pathology, which is in line with studies from the pertinent literature.

Patients with benign PTB do not require postoperative adjuvant therapy. Although the usefulness of postoperative chemotherapy for patients with malignant and borderline PTB is debatable, the consensus is that chemotherapy offers no appreciable advantages to patients with PTB. Radiation therapy has been shown in certain studies to decrease the local recurrence in patients with borderline and malignant PTB; nevertheless, opinions on whether postoperative patients require radiation therapy are divided.^21^ Breast‐conserving surgery was performed on 46 patients with borderline and malignant PTB in a prospective multicenter trial conducted by Brath et al.^22^ Adjuvant radiation enhanced local control significantly after guaranteeing negative margins, and after a mean follow‐up of 50 months, no local recurrence was identified in any of the patients. The available data suggest that patients with borderline and malignant PTB who have breast‐conserving surgery and for whom negative margins are difficult to acquire may benefit from postoperative adjuvant radiation therapy.^23^, ^24^

## 4 CONCLUSION

Although patients with PTB have a fair prognosis, there is a significant chance of metastasis and recurrence, particularly in those whose tumors are larger than 5 cm. As soon as the diagnosis is made, surgery should be carried out, and other adjuvant treatment measures should be applied in accordance with the patient's particular circumstances to reduce the chance of recurrence. Given the variety of PTB recurrence risks and biological characteristics, it is necessary to conduct a long‐term follow‐up of the patients after surgery.

## AUTHOR CONTRIBUTIONS

**Xiaoxiao Dong:** Investigation; methodology; validation; visualization; writing – original draft; writing – review and editing. **Dong Song:** Conceptualization; supervision; validation; visualization; writing – original draft; writing – review and editing. **Jinxiu Ma:** Data curation; investigation; resources. **Jian Sun:** Investigation; writing – review and editing. **Xiaozhen Wang:** Data curation; funding acquisition; supervision; validation; writing – review and editing.

## FUNDING INFORMATION

The study was funded by National Natural Science Youth Foundation (81602335), The Education Department of Jilin Province (JJKH20241325KJ), Natural Science Foundation of Jilin Province (20200201423JC), and Wu Jieping Medical Foundation (320.6750.2023‐03‐35).

## CONFLICT OF INTEREST STATEMENT

All authors state that they have no competing interests.

## ETHICS STATEMENT

The study obtained the approval of the Ethics Committee of The First Hospital of Jilin University (ethics number: AF‐IRB‐032‐06).

## CONSENT

Written informed consent was obtained from the patient to publish this report in accordance with the journal's patient consent policy.

## Data Availability

All data generated or analyzed during this study are included in this article.
